# Supplementary material for: Small-rotative fixed-target serial synchrotron crystallography (SR-FT-SSX) for molecular crystals
Source: Commun Chem. 2024 Nov 13;7:264. doi: 10.1038/s42004-024-01360-7 (PMC11561299; doi:10.1038/s42004-024-01360-7)
Supplement: Supplementary file 2 — Supplementary Material [file 42004_2024_1360_MOESM2_ESM.pdf]

Small Rotative Fixed-target Serial Synchrotron Crystallography (SR-FT-SSX) for Molecular Crystals

Supplementary Information

Sam G. Lewis,[1,2] Ben A. Coulson,[1] Anna J. Warren,[2] Mark R. Warren*[2] & Lauren E. Hatcher*[1]

[1] School of Chemistry, Cardiff University, Park Place, Cardiff, CF10 3AT

[2] Diamond Light Source, Harwell Science and Innovation Campus, Fermi Avenue, Didcot, Oxfordshire, OX11 0DE

Supplementary Note 1: Sample preparation for grid loading

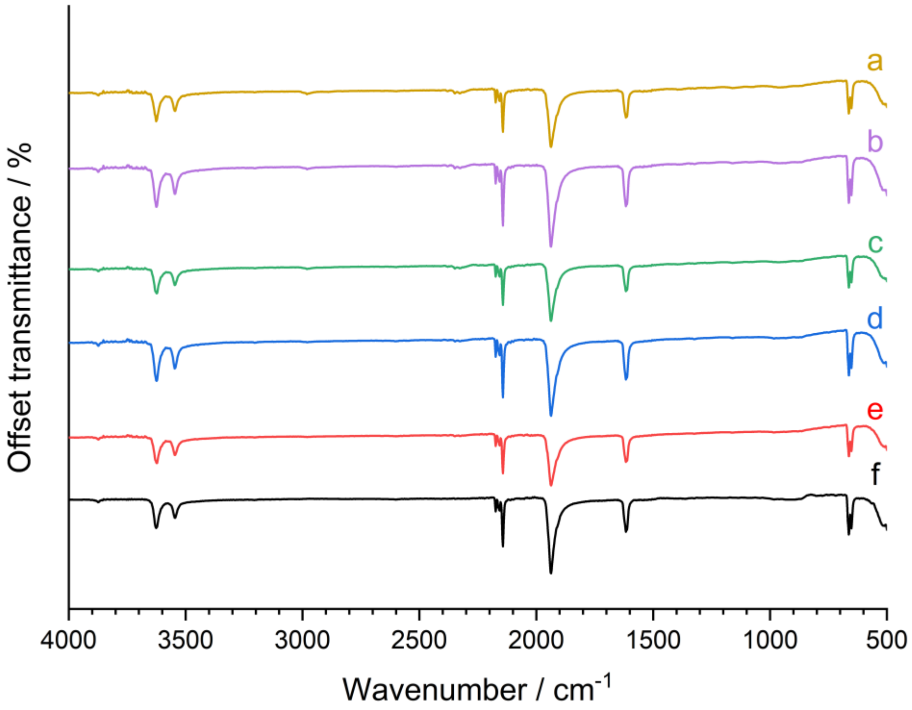

Figure S1. FT-IR spectra of microcrystal batches recovered after suspension in the following antisolvents: a) 1-butanol, b) benzyl alcohol, c) chloroform, d) diethyl ether compared to e) a sample from the acetonitrile mixture and f) SNP.2H_2O obtained from a ground sample from the stock bottle. The data confirm that no change in the crystal form is observed by suspending the microcrystals in any of the tested antisolvents.

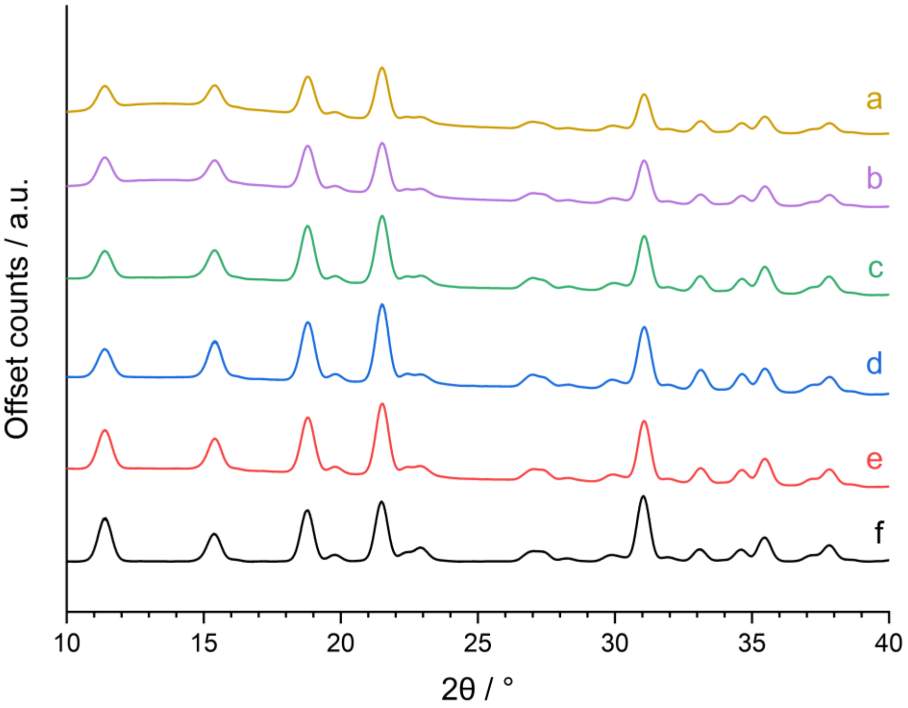

Figure S2. PXRD patterns of microcrystal batches recovered after suspension in the following antisolvents: a) 1-butanol, b) benzyl alcohol, c) chloroform, d) diethyl ether compared to e) a sample from the acetonitrile mixture and f) SNP.2H_2O

obtained from a ground sample from the stock bottle. The data confirm that no change in the crystal form is observed by suspending the microcrystals in any of the tested antisolvents.

Supplementary Note 2: Sample preparation (grid loading)

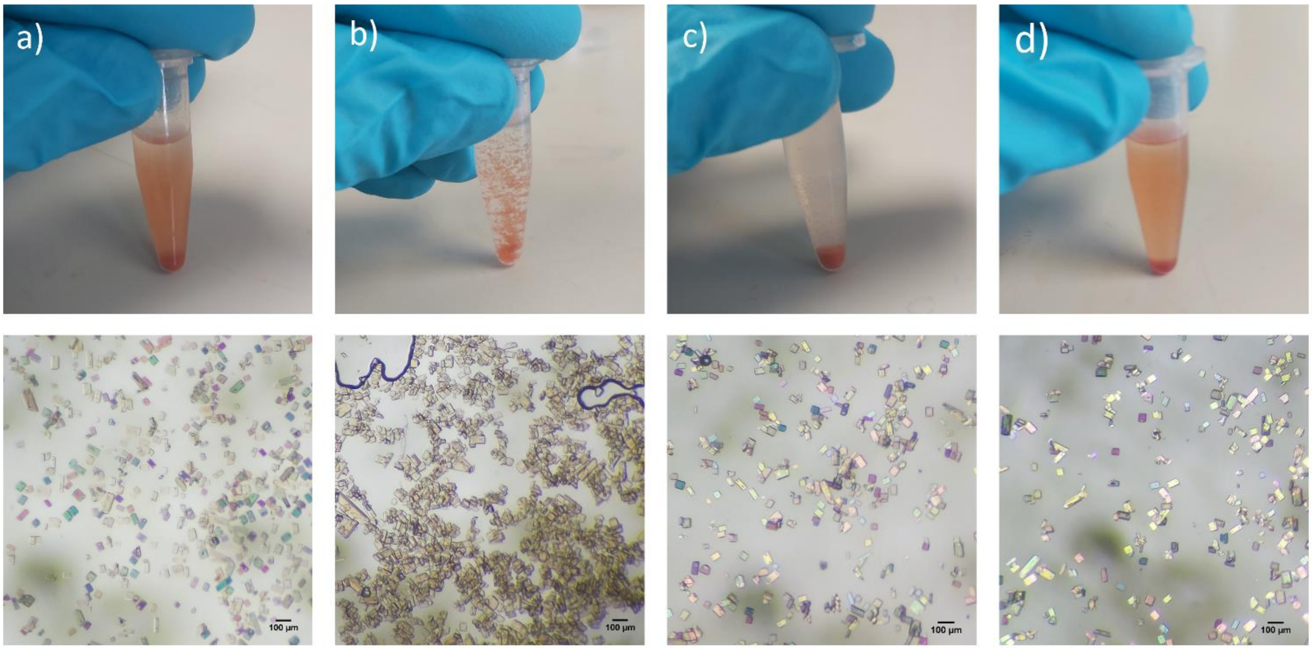

Figure S3. Images of SNP.2H_2O crystals suspended in various antisolvents: a) benzyl alcohol, b) chloroform, c) diethyl ether, and d) 1-butanol taken 5 seconds after agitating (top row) and polarized optical micrographs of a small aliquot of crystals pipetted onto a microscopy slide. The images show that 1-butanol provided the most even suspension of crystals for distribution onto the fixed-target silicon nitride serial grids.

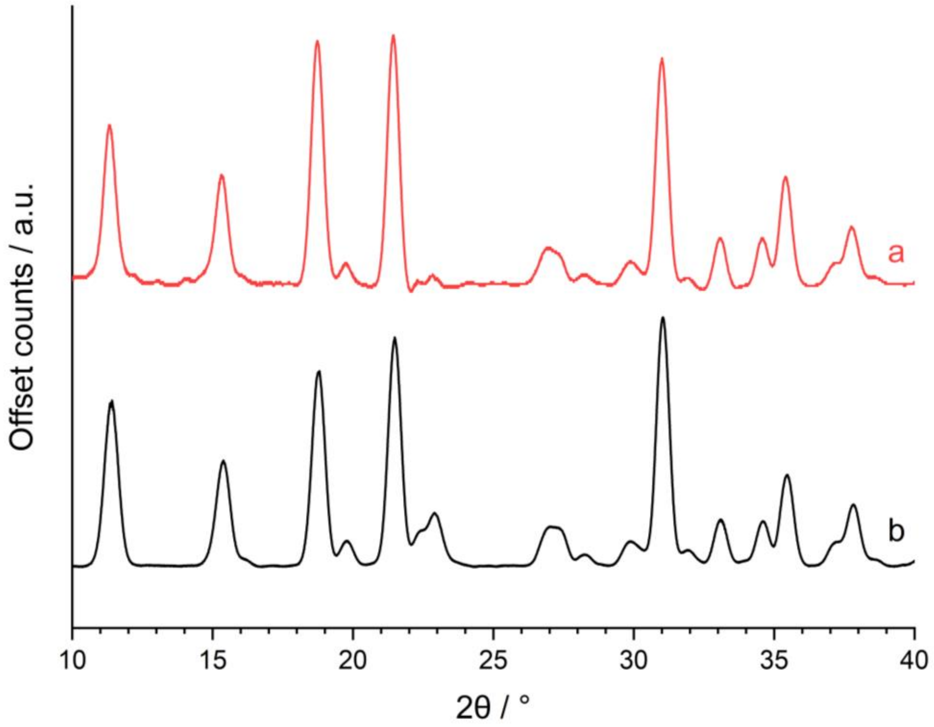

Figure S4. PXRD of a) a recovered SNP.2H_2O microcrystal batch following suspension in 1-butanol for a period of 6 months compared to b) SNP.2H_2O obtained from a ground sample from the stock bottle.

Method for creating the 1-butanol suspension for sample delivery onto the grids

To create the 1-butanol suspension, a microcrystal batch in the mother liquor was transferred to a 1.5 mL microcentrifuge tube and the crystals allowed to settle naturally, before the supernatant was carefully removed by pipette. 1 mL of 1-butanol antisolvent was then added to the crystals and the tube was agitated to produce an even suspension. This produced a crystal suspension of 0.16 g/g, from which a series of concentrations were produced by serial dilution (0.08, 0.04, 0.02 g/g) which were trialed for the best sample loadings. Preparation of a crystal suspension of SNP.2H_2O by this method was sufficiently stable to prepare grids either in the home-lab or following transportation to Diamond Light Source.

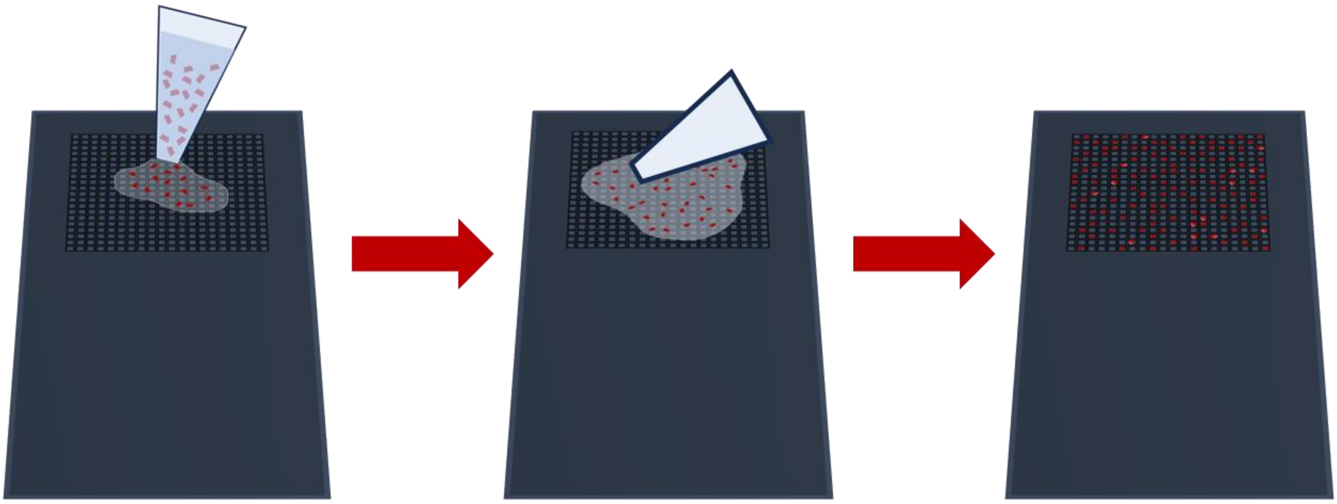

Figure S5. Graphical diagram to illustrate the sample loading procedure onto the fixed-target serial chip by pipetting 5 µL of the SNP stock solution onto the surface of the grid, before spreading with the pipette tip and removing the 1-butanol by vacuum filtration.

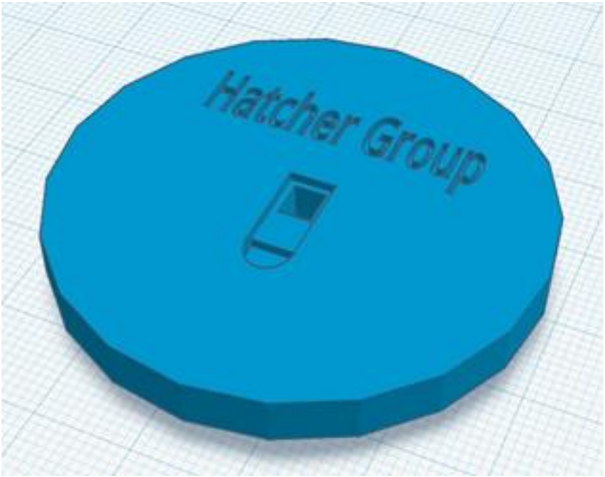

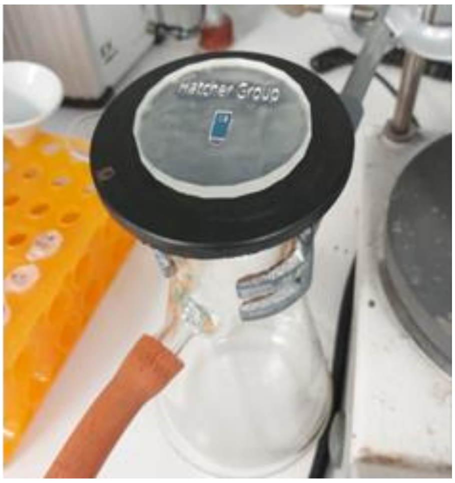

Figure S6. Images of the bespoke 3D-printed resin puck used for sample loading of the fixed-target serial grids.

Supplementary Note 3: Data Collection

Serial crystallography experiments were performed on the Small Molecule Crystallography Beamline I19 at Diamond Light Source, using a dual air-bearing fixed-χ diffractometer equipped with a Dectris Pilatus 2M pixel-array photon-counting detector in Experiment Hutch 1 (EH1). Three linear piezo stages from PM-Bearings (RTP-1510-0.1 micron) are mounted on the air-bearing phi (φ) axis, which has a 40 °/s rotation speed. To improve the indexing success rate, the number of reflections per image were maximized by using high-energy X-rays, which also provided high diffraction resolution (> 0.6 Å) in a single detector position. The X-ray wavelength was tuned to the Ag edge (0.4859 Å, 25.5140 keV) and a detector distance of 160 mm was used. All data collections were conducted at room temperature (RT, 20°C).

The 400-well grid was loaded with SNP.2H_2O microcrystals, as described above, and mounted on the diffractometer using a modified magnetic base manually orientated to be approximately normal to the X-ray beam path and on-axis viewing system. A custom Fixed Target Serial Crystallography collection graphical user interface (GUI) (Figure S7), developed on Beamline I19, was implemented to enable user-friendly alignment and set up of the data collection.

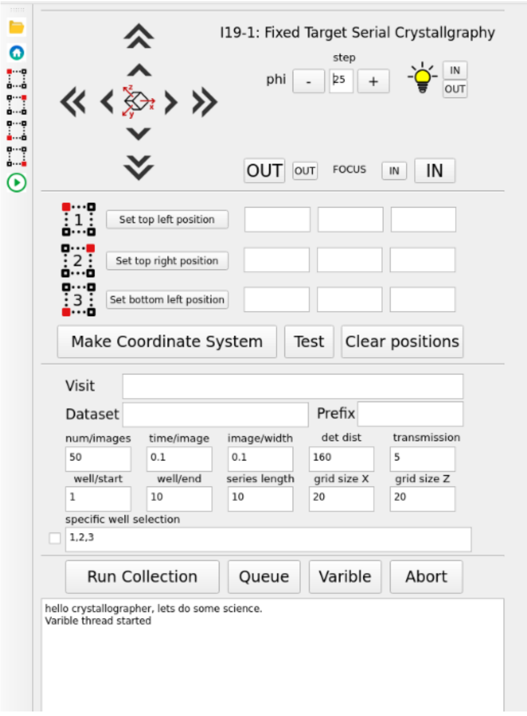

Figure S7. GUI developed for set-up and collection of SR-FT-SSX datasets on Beamline I19-1 at Diamond Light Source.

Collecting small wedges on a fixed-target grid requires determination of the coordinates (sample stage x, y, and z), in reference to the diffractometer center of rotation, at each grid position. Once these coordinates are established, the sample stage can be precisely moved to the desired well, allowing the crystal within that well to be rotated around the φ axis without precessing in or out of the X-ray beam.

First, the top-left well is visually aligned in the sample stage x-z plane using the on-axis viewing camera. The y position (focus) is located by rotating the φ axis by 25° and then adjusting the sample stage y-axis to bring the well back to the center of the rotation crosshair. The coordinates are saved in the collection GUI, and this process is repeated for the top-right and bottom-left wells. The x, y and z coordinates for all other grid positions can then be calculated based on these three reference points.

Data collection follows a traditional "snake scan" pattern: starting from the top-left corner, the scan proceeds left to right across the first row of the serial chip, then shifts down to the next row and moves right to left, and so on (see main manuscript Figure 2c) across the total 400 wells. Diffraction images are taken over a 5° rotation at each crystal (50 images total, 0.1° image width). To minimize overhead times, the rotation of the φ-axis is alternated between forward and backward directions from one well to the next, eliminating the need to return to the starting φ position

for each collection. Additionally, to avoid significant overheads from starting and stopping the Pilatus detector for each well, a multi-trigger collection mode is employed so that data is collected from multiple wells in a single sequence.

Supplementary Note 4: Data Processing

This Note provides an overview of the procedures for data processing on SR-FT-SSX data collected for small molecule systems as utilized in this publication. This was achieved via a series of scripts that were written specifically for the target system SNP.2H_2O and SR-FT-SSX experiments conducted in Experimental Hutch 1 (EH1) on Beamline I19 at Diamond Light Source. However, these scripts may serve as a starting template for others to conduct similar experiments.

One of the biggest challenges in the processing of SR-FT-SSX data is handling the large number of datasets produced, in this instance 400 per collection. Though automation is essential when handling such a large quantity of data, we recommend first visually inspecting the frames produced during data collection and manually processing a small number of wells through DIALS. This initial assessment step helps to identify what necessary parameters may be required, which can then be directly applied to the batch processing scripts.

Chipreader.py script

Chipreader.py automatically generates and executes the required input scripts for the software Xia2 to process the partial datasets from all 400 wells through DIALS,[1] producing partial structures from each well occupied by a suitable crystal. The mandatory inputs are the path to the data collection directory and the mode of indexing to be conducted, which for this study was either:

i) a real_space_grid_search approach, where the user is also required to provide the known unit cell parameters and space group (Route A in the main manuscript), or
ii) a 3-dimensional fast Fourier transform (fft3d) approach, used for autoindexing and ab initio determination of unit parameters (Route B in the main manuscript).

	A	B	C	D	E	F	G	H	I	J	K	L	M	N	O	P	Q	R	S	T	U	V	W	X
1	wellnu	jobnum	solve	loadstatu	highreslim	lowreslim	completenes	multiplicit	isigm	Rmerg	Rme	Rpim	a_length	b_length	c_length	alpha	beta	gamma	ccha	totalob	totaluniqu	numindexe	numunindexe	percentindexe
101	100	7312406	True	solvable	1.12	3.18	9.9	1	3.4	0	0	0	6.200(3)	11.892(4)	15.572(5)	90	90	90	0	51	51	44	14	75
102	101	7312407	0	0	0	0	0	0	0	0	0	0	0	0	0	0	0	0	0	0	0	0	0	0
103	102	7312408	0	0	0	0	0	0	0	0	0	0	0	0	0	0	0	0	0	0	0	0	0	0
104	103	7312409	True	solvable	0.59	4.75	11.9	1	13.2	0.012	0.017	0.012	6.1978(4)	11.8951(4)	15.5595(4)	90	90	90	1	411	395	312	231	57
105	104	7312410	0	0	0	0	0	0	0	0	0	0	0	0	0	0	0	0	0	0	0	0	0	0
106	105	7312411	0	0	0	0	0	0	0	0	0	0	0	0	0	0	0	0	0	0	0	0	0	0
107	106	7312412	True	solvable	0.59	3.34	5.9	1.1	10.5	0.021	0.029	0.021	11.8992(5)	6.2008(3)	15.563(5)	90	90.000(5)	90	0.999	410	375	264	50	84
108	107	7312413	True	solvable	0.6	5.95	5.6	1.2	13	0.069	0.097	0.069	11.8986(5)	6.1997(3)	15.558(5)	90	90.001(3)	90	0.989	416	341	291	254	53
109	108	7312414	True	solvable	0.58	9.45	11.8	1	9.1	0.015	0.021	0.015	6.20133(19)	11.8973(4)	15.5577(9)	90	90	90	1	420	409	312	357	46
110	109	7312415	True	solvable	0.77	3.31	16	1	4.4	0.032	0.045	0.032	6.1988(7)	11.8947(18)	15.5607(9)	90	90	90	0.998	253	244	133	14	90
111	110	7312416	True	solvable	0.92	4.97	4.7	1	3.8	0	0	0	13.427(19)	15.563(4)	22.074(2)	89.984(11)	95.06(3)	89.98(8)	0	581	581	343	599	36
112	111	7312417	True	solvable	0.58	5.95	9.1	1.3	12.3	0.054	0.076	0.054	6.2002(3)	11.8989(4)	15.566(4)	90	90	90	0.989	414	317	284	304	48
113	112	7312418	True	solvable	0.59	5.5	6.6	1	15.5	0	0	0	11.8940(11)	6.2001(2)	15.5601(5)	90	90.008(5)	90	1	422	419	335	299	52
114	113	7312419	True	solvable	0.59	5.18	6.4	1	8.8	0.009	0.013	0.009	11.8932(17)	6.1999(3)	15.560(2)	90	90.00(2)	90	1	403	391	264	429	38
115	114	7312420	True	solvable	0.57	5.5	11.3	1	14	0.003	0.005	0.003	6.1997(2)	11.8984(3)	15.5640(6)	90	90	90	1	417	407	310	257	54
116	115	7312421	True	solvable	0.63	4.49	14.2	1	6.6	0.01	0.014	0.01	6.2001(3)	11.8957(5)	15.5612(7)	90	90	90	1	395	384	213	119	64
117	116	7312422	True	solvable	0.59	5.18	5.9	1	12.6	0.006	0.008	0.006	6.20043(16)	11.8950(5)	15.5578(16)	90	89.981(4)	90	1	371	361	287	16	94
118	117	7312423	0	0	0	0	0	0	0	0	0	0	0	0	0	0	0	0	0	0	0	0	0	0
119	118	7312424	0	0	0	0	0	0	0	0	0	0	0	0	0	0	0	0	0	0	0	0	0	0
120	119	7312425	0	0	0	0	0	0	0	0	0	0	0	0	0	0	0	0	0	0	0	0	0	0
121	120	7312426	0	0	0	0	0	0	0	0	0	0	0	0	0	0	0	0	0	0	0	0	0	0
122	121	7312427	0	0	0	0	0	0	0	0	0	0	0	0	0	0	0	0	0	0	0	0	0	0
123	122	7312428	0	0	0	0	0	0	0	0	0	0	0	0	0	0	0	0	0	0	0	0	0	0
124	123	7312429	0	0	0	0	0	0	0	0	0	0	0	0	0	0	0	0	0	0	0	0	0	0
125	124	7312430	True	solvable	0.6	9.45	10.7	1	12.5	0.007	0.01	0.007	6.1998(2)	11.8982(3)	15.5609(6)	90	90	90	1	353	345	302	27	91
126	125	7312431	0	0	0	0	0	0	0	0	0	0	0	0	0	0	0	0	0	0	0	0	0	0
127	126	7312432	0	0	0	0	0	0	0	0	0	0	0	0	0	0	0	0	0	0	0	0	0	0
128	127	7312433	0	0	0	0	0	0	0	0	0	0	0	0	0	0	0	0	0	0	0	0	0	0
129	128	7312434	0	0	0	0	0	0	0	0	0	0	0	0	0	0	0	0	0	0	0	0	0	0
130	129	7312435	True	solvable	0.61	5.5	7.1	1	4.9	0	0	0	15.579(3)	6.2005(3)	23.7957(14)	90	90.039(12)	90	1	822	818	315	458	40

Figure S8. An example section of the .CSV files generated by chipreader.py containing structure quality descriptors for the wells collected during the SR-FT-SSX experiment.

By running Chipreader.py, the user submits each partial dataset collected on the individual crystal through a routine DIALS processing regime, which is treated as if it is a standard (though incomplete) single crystal X-ray data collection. Thus, each partial dataset is first subject to spot (reflection) finding, indexing (through the specified mode of indexing as described above), integration and scaling procedures.

Once Chipreader.py has finished with the processing of all of the wells a .CSV file is generated, populated with the structure quality descriptors for the wells that were successfully processed through DIALS, as shown in Figure S8. The user is then able to assess the data and filter based on their chosen structure quality descriptors, such as:

- High resolution limit
- Low resolution limit
- Completeness
- Multiplicity
- $I/\sigma(I)$
- $R_{\mathrm{merge}}(I)$
- $R_{\mathrm{meas}}(I)$
- $R_{\mathrm{pim}}(I)$
- $CC_{1/2}$
- Total observations
- Total unique observations
- Unit cell parameters
- Crystal system
- Space group

Three criteria were used to filter the data in this work: percentage of spots indexed, $I/\sigma(I)$, and R_{pim}. For distributions of these parameters across samples in the serial grid see Figure S9. Only wells with datasets exceeding these quality criteria were scaled and merged to produce the final combined structures.

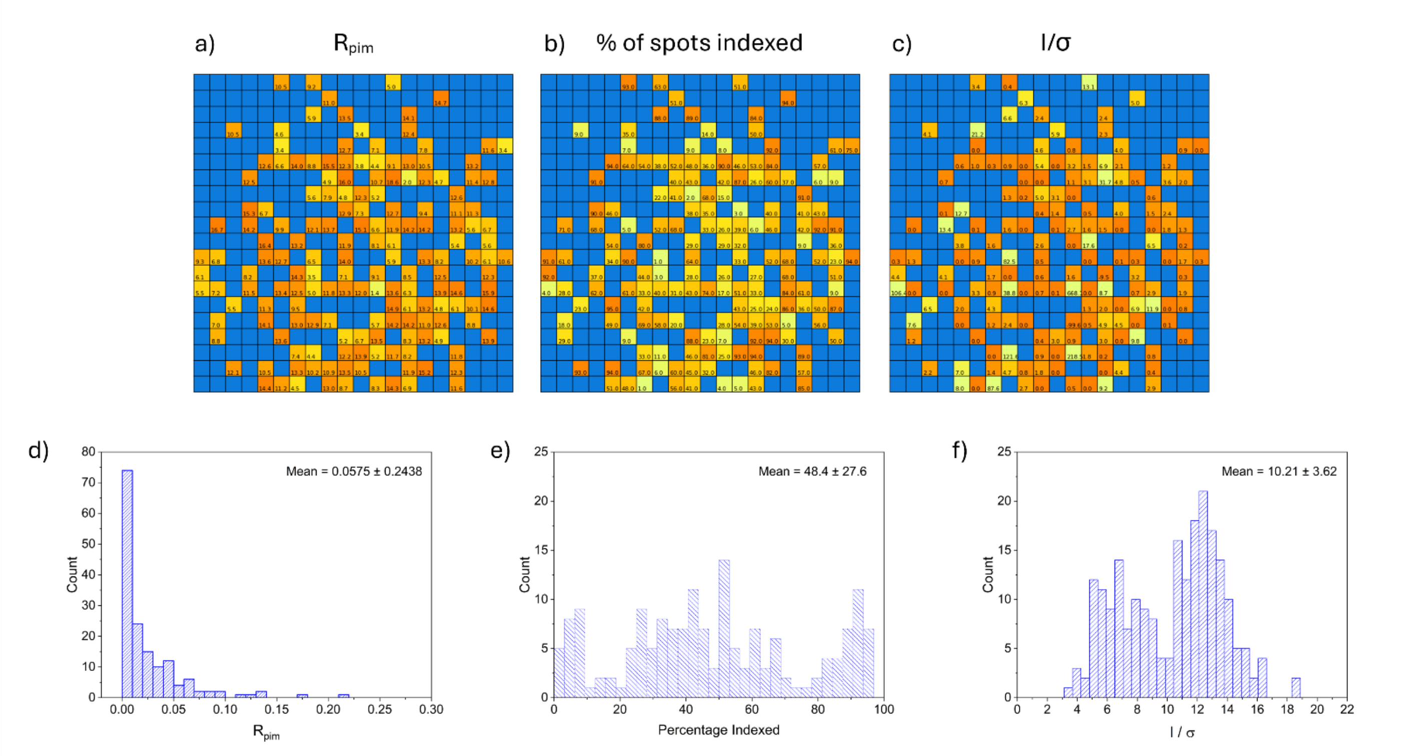

Figure S9. Visual representations of the fixed-target grid well positions in real space mapped to their dataset quality factor values i.e. selection criteria used to select the most successful partial datasets) in the iterative processing route used to generate structure **1**: a) R_{pim} (yellow = high (poor) value, orange = low (good) value, blue = no value/crystal), b) % of spots indexed (yellow = low (poor) % value, orange = high (good) % value and blue = no value/crystal) and c) $I/\sigma(I)$ (yellow = low (poor) value, orange = high (good) value, blue = no value/crystal), alongside histogram representations of the structure quality factors d) R_{pim}, e) % of spots indexed and f) $I/\sigma(I)$.

For SNP.2H_2O, the percentage of spots indexed was selected as the first metric of choice, with a high value indicating a singly occupied well. $I/\sigma(I)$ was also selected as it illustrates the strength of diffraction and represents the reliability of

the data. A third data filtering step was carried out using R_{pim} as a structure quality factor. For details on each of the available structure quality factors, see Supplementary Note 5.

The limits of the percentage of spots indexed (50%), $I/\sigma(I)$ (6.0) and R_{pim} (5%), were selected via an iterative process to maximise the number of datasets selected for merging, without reducing overall data quality. Using these cutoff values for the data produced by indexing via fft3d and real_space_grid_search gave 28 and 66 datasets, respectively, which were used to generate crystal structures **1** and **2**, as discussed in the manuscript. For the purposes of this work, the limits were kept consistent between the two structures to allow direct comparison of the two indexing methods. It is noted that further improvements on merged data quality could be achieved by tweaking the limits on the chosen criteria, but this was not carried out as the resulting merged data was of sufficiently high quality.

Scaling data, structure solution and refinement

The merged dataset is produced by calling the DIALS.scale command towards the selected partial datasets from the chosen wells, which configures a “Multiscaler” regime to scale the individual datasets being merged to account for any variation in the diffraction data due to experimental factors such as crystal-to-crystal size variations or fluctuations in the synchrotron beam. Scaling of multiple datasets from different crystals is possible for our method because within our partial datasets collection on individual crystals we have sufficient coverage of reciprocal space, and good sampling of the reflection profiles due to our thin-slicing rotative approach. An example output from the Multiscaler merging process is archived with the data processing scripts online and can be accessed via the Figshare link provided in the Data Availability statement. Once complete, standard SHELX .INS and .HKL files are output that can be reprocessed through a space group determination program of choice. Following this, a unit cell refinement is conducted on the scaled dataset to provide estimated standard deviations. The final data is then exported in SHELX format for structure solution and refinement. In this publication, the final structures were solved with SHELXT[2] and refined by full matrix least squares on F^2 using SHELXL[3] via the Olex2 GUI.[4] Hydrogen atom positions were determined from the Fourier electron density difference map, then refined using a riding model. The hydrogen atom isotropic displacement parameters were fixed to $U_{\mathrm{iso}}(H)$= 1.2 × the parent oxygen atom.

Use of SR-FT-SSX to study unknown systems

To illustrate the capability and power of the SR-FT-SSX to study unknown crystal systems, the same data has been processed via two representative processing pathways, as outlined in the SR-FT-SSX workflow (Scheme 1). To this effect we generated structures **1** and **2** in an iterative fashion. Following grid loading and data collection in steps 1 and 2, the data was first processed with the script chipreader.py by selecting to use the autoindexing mode of route B. Using the fft3d method all 400 wells have been indexed and integrated through xia2 in step 3. From the wells that successfully indexed and integrated, the unit cell parameters, crystal system and space group found are then collated into a .CSV file as per Figure S8.

Data is then filtered to determine the most commonly occurring unit cell parameters, crystal system, and space group. Of the 166 wells which could be indexed by fft3d, 103 had the correct unit cell parameters for SNP.2H_2O. By merging correctly indexed wells which also satisfied the chosen R_{pim}, % of spots indexed, and I/σ cutoffs, a good quality crystal structure **1** was then generated, albeit with a low overall completeness (95.3%). Full parameters for **1** are listed in Table 1. Although DIALS mistakenly determined the space group of **1** to be *Pmmm*, upon solving the combined structure with SHELXT the correct space group *Pnnm* was found.

A further refined structure **2** was then obtained by reprocessing the data (route A in the flow diagram) using the parameters obtained from structure **1**. The use of real space grid search indexing proved powerful for increasing the number of datasets available for data scaling and merging for structure **2**, with 221 datasets being indexed which all had the correct unit cell parameters, more than doubling the number of datasets obtained without providing any known cell parameters. This proves the validity of using this iterative, 2-step approach to generate the best possible combined final structure. Visual comparisons of the spread of data obtained across the grid by both fft3d and real space grid search can be seen in Figures S9 and 3.

Comparisons of the spread of experimentally determined unit cell parameters and volumes can be seen in Figures S10 and S11. The unit cell parameters determined by real space grid search were incredibly well defined at the correct values for sodium nitroprusside (*a*=6.2, *b*=11.9, *c*=15.6, *α*=90, *β*=90, *γ*=90). The highest frequency unit cell parameters found by fft3d were also the correct values, however while the c length was largely only the correct value with a few outliers the *a* and *b* lengths were occasionally determined in the wrong order, resulting in the bimodal distribution of both dimensions in Figure S10.

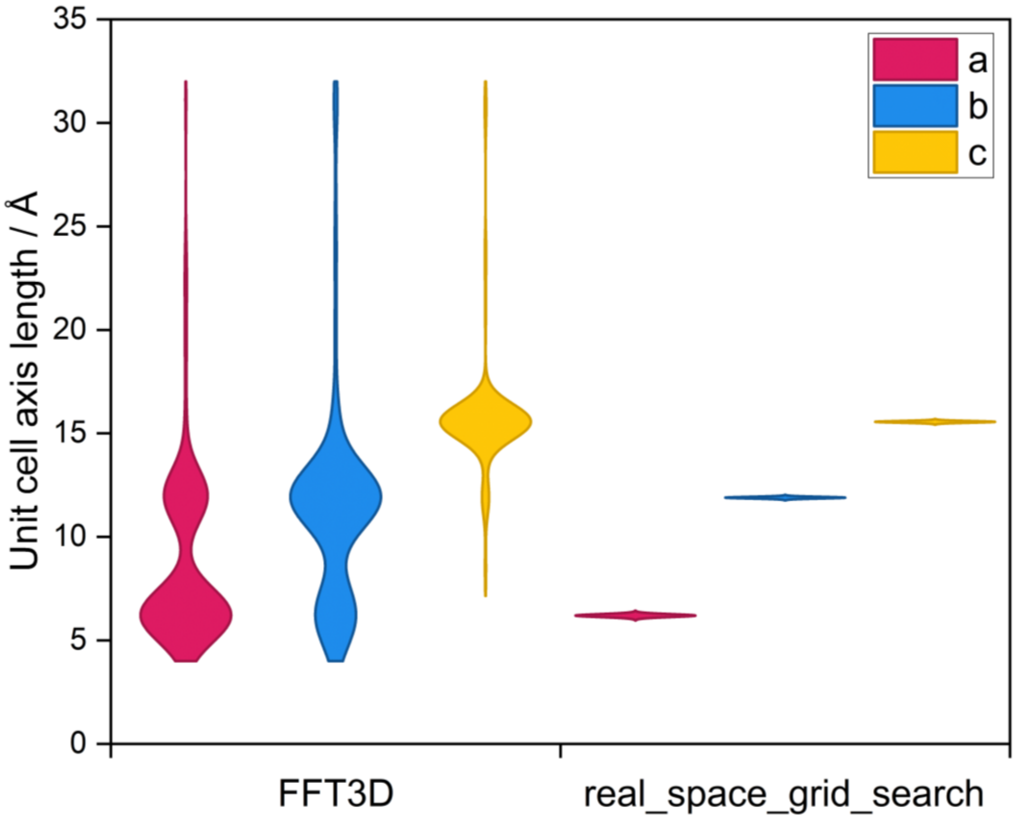

Figure S10. Histograms illustrating the distribution of unit cell parameters indexed during the data processing of **1** and **2** by 3D fast Fourier transform or real space grid searching methods respectively.

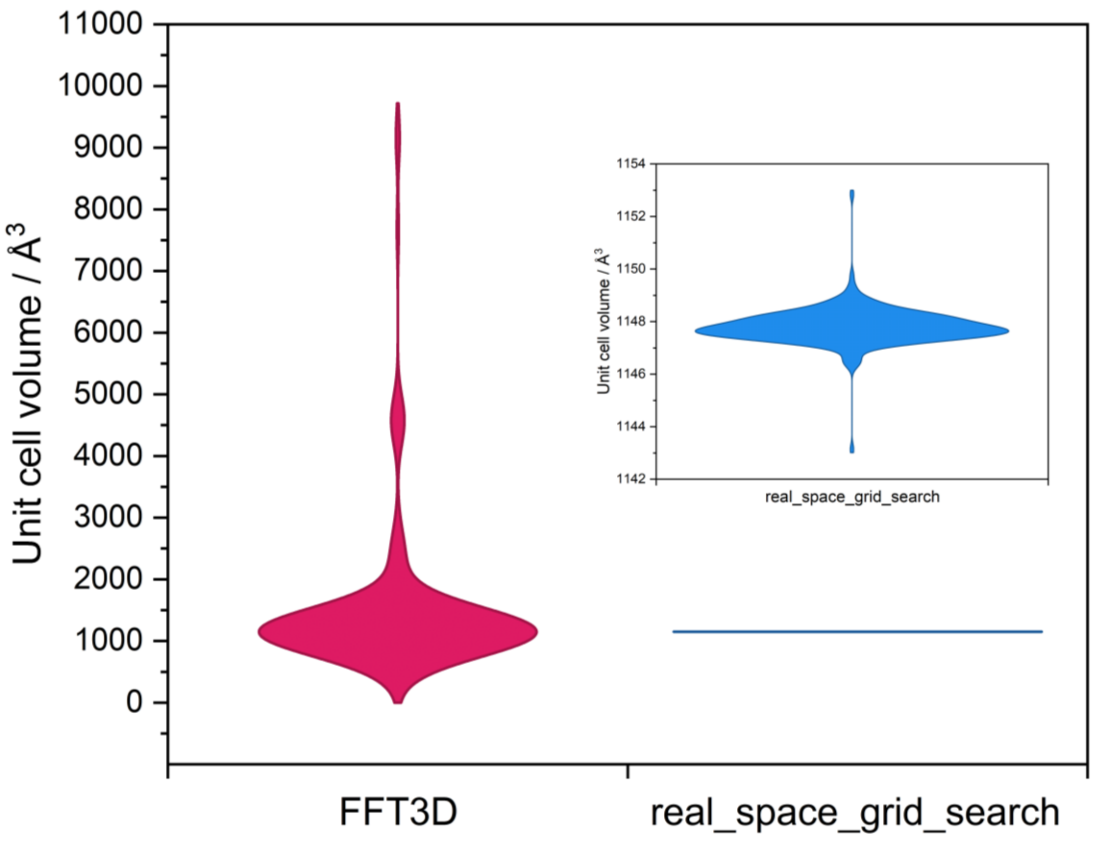

Figure S11. Histograms illustrating the distribution of volumes found during the data processing of **1** and **2** by 3D fast Fourier transform or real space grid searching methods respectively.

Single crystal dataset information	
Empirical formula	$Na_2[FeC_5N_6O]\cdot 2H_2O$
Formula weight	297.95
Wavelength (Å)	1.5
Temperature / K	294.4
Crystal system	orthorhombic
Space group	*Pnnm*
a (Å)	6.2035(3)
b (Å)	11.8984(5)
c (Å)	15.5536(7)
Volume (Å^3)	1148.04(9)
Z	4
ρ_{calc}g/cm^3	1.724
μ/mm-1	11.387
F(000)	592.0
Crystal size/mm^3	0.06 x 0.04 x 0.02
Total reflections	3995
Unique reflections	1231
Mean $I/\sigma(I)$	28.1
Redundancy	3.6
R_1 (%) [$I>2\sigma(I)$]	0.0279
wR_2 (%) [all data]	0.0726
GooF on F^2	1.072
Completeness (%)	99.3
Largest peak/hole (eÅ^{-3})	0.29/-0.32

Table S1. Crystal data for a representative SNP single crystal from the produced microcrystal batch collected on an in-house RIGAKU Synergy-R diffractometer.

Supplementary Note 5: Structure Quality Factors

It was not trivial to select a structure quality factor to assess overall data quality. Assessment of data quality is available through multiple *R*-factors (R_{merge}, R_{meas}, and R_{pim}) or alternatively $CC_{1/2}$ which is routinely used as a structure quality factor of choice in for macromolecular crystals. The equations to calculate R_{merge}, R_{meas}, R_{pim}, and $CC_{1/2}$ are shown in equations S1 – S4.

$$R_{merge} = \frac{\sum_{hkl}\sum_{i}^{n}\left|I_i - \bar{I}\right|}{\sum_{hkl}\sum_{i}^{n} I_i}$$

Equation S1. The equation to calculate the precision of unmerged data indicator *R*-factor R_{merge}.[5]

$$R_{meas} = \frac{\sum_{hkl}\sqrt{\frac{n}{n-1}}\sum_{i}^{n}\left|I_i - \bar{I}\right|}{\sum_{hkl}\sum_{i}^{n} I_i}$$

Equation S2. The equation to calculate the unbiased unmerged precision indicator *R*-factor R_{meas}.[5]

$$R_{pim} = \frac{\sum_{hkl}\sqrt{\frac{1}{n-1}}\sum_{i}^{n}\left|I_i - \bar{I}\right|}{\sum_{hkl}\sum_{i}^{n} I_i}$$

Equation S3. The equation to calculate the merged precision *R*-factor indicator R_{pim}.[6]

$$CC_{1/2} = \frac{1}{1 + \alpha R_{\mathrm{diff}}^2}$$

Equation S4. The equation to calculate the Pearson correlation coefficient $CC_{1/2}$.[7]

$CC_{1/2}$ is typically chosen as the metric of choice in macromolecular crystallography (MX) as it is able to detect weak signals typically present in higher resolution shells of crystallographic data for protein crystals. $CC_{1/2}$ values are calculated for thin resolution shells to observe their dependence with increasing resolution. $CC_{1/2}$ is calculated by considering R_{diff} which is defined as the average fractional intensity difference of two half datasets consisting of two non-overlapping equally sized sets of the measurements taken for each reflection.[7] In general, protein crystals are poorer diffractors than small molecule crystals and their diffraction pattern does not achieve the same resolution. The high resolution cut-off for the SNP.2H_2O crystals used in this study is d_{min} = 0.6 Å and there remains suitably strong diffraction intensity even at this resolution, thus $CC_{1/2}$ is not observed to deviate significantly from 1, as shown in Figure S8. We therefore decided that this metric was not informative for our crystals and instead referred to other *R*-factors.

R_{meas} is not considered as the values are typically greater than those obtained for R_{pim} and R_{merge}. R_{pim} was favoured as it takes into account an increase in precision with the square root of the number of independent reflections.[6] It is important to note however that all *R*-factors become less meaningful upon merging and scaling of multiple datasets of varying quality. This is due to the fact that each reflection is weighted equally rather than with respect to the quality of the data.[8] However, as our crystallization approach generates highly consistent microcrystal batches of SNP.2H_2O with a narrow size distribution and consistent plate-like habit (see Figure 1, main manuscript), we did not observe significant variability in the diffraction quality obtained across the fixed-target grid. Thus, we are confident that our use of R_{pim} as a structure quality factor is valid in this case. Furthermore, by filtering the data according to multiple structure quality factors (including signal-to-noise, $I/\sigma(I)$, and % of spots indexed, as well as R_{pim}) we aim to account for the shortcomings of individual metrics and increase the overall quality of the final merged crystal structure.

Supplementary Note 6: Unit Cell Parameters from Fewer Numbers of Frames

For the purposes of achieving higher quality crystal structures with greater completeness, structures **1** and **2** were processed using all 50 frames collected over a 5° wedge at each well position that was selected according to the structure quality factors, as described in the main manuscript. However, further investigation confirmed that this large a number of frames is not always required to enable indexing of the SNP.2H_2O unit cell via either the 3-dimensional fast Fourier transform (fft3d, enabling ab initio determination of unit cell parameters, Route B in the manuscript) or the real space grid search methods (requiring the known unit cell parameters to be input at the start of processing, Route A in the manuscript). Test data processing runs with the Route B, fft3d approach showed that reasonable unit cell parameters could be determined ab initio from as few as 3 frames when the data were processed through DIALS manually, although this was limited to grid wells that produced strong diffraction with a high % of spots matching the indexed unit cell (i.e. indicating an individual single crystal was occupying that well). To illustrate this capability, a strongly-diffracting crystal (grid well position 025), with a very high value of % spots indexed, was processed under various different conditions:

i) All 50 frames, processed via the automated pipeline using the fft3d indexing method (A-FFT3D), Route B
ii) All 50 frames, processed manually through DIALS using the fft3d indexing method (M-FFT3D), Route B
iii) 10 frames only, processed manually through DIALS using the fft3d indexing method (M-FFT3D), Route B
iv) 5 frames only, processed manually through DIALS using the fft3d indexing method (M-FFT3D), Route B
v) 3 frames only, processed manually through DIALS using the fft3d indexing method (M-FFT3D), Route B
vi) All 50 frames, processed via the automated pipeline using the real-space grid search indexing method, with known unit cell parameters provided (A-RSGS), Route A
vii) All 50 frames, processed manually through DIALS using the real-space grid search indexing method, with known unit cell parameters provided (A-RSGS), Route A
viii) Only 3 frames, processed manually through DIALS using the real-space grid search indexing method, with known unit cell parameters provided (A-RSGS), Route A

The results are summarized in Table S2 below and the corresponding outputs from the DIALS processing software are included in Figures S12-19. Table S2 confirms that reasonable unit cell parameters can be determined from all of the processing routes tested, although it is clear that the accuracy and reliability of the unit cell parameters decreases as the number of frames used also decreases.

Indexing method	Number of frames	Number of reflections	Unit cell parameters					
			a / Å	*b* / Å	*c* / Å	α / °	β / °	γ / °
A-FFT3D	50	289	6.20119(11)	11.8969(3)	15.5632(9)	90.007(3)	90.014(4)	89.9929(16)
M-FFT3D	50	306	6.20113(10)	11.8969(3)	15.5629(9)	90.000(3)	89.986(3)	89.9930(15)
M-FFT3D	10	65	6.2008(2)	11.8977(7)	15.562(2)	90.017(9)	89.986(7)	89.988(4)
M-FFT3D	5	41	6.2021(5)	11.8989(13)	15.559(4)	89.965(18)	89.990(15)	90.005(6)
M-FFT3D	3	24	6.2008(7)	11.891(2)	15.554(6)	90.05(3)	89.91(3)	90.004(10)
A-RSGS	50	289	6.20151(11)	11.8976(3)	15.5647(9)	90.0	90.0	90.0
M-RSGS	50	306	6.20144(10)	11.8976(3)	15.5647(9)	90.0	90.0	90.0
M-RSGS	3	24	6.2067(7)	11.902(2)	15.593(6)	90.0	90.0	90.0

Table S2. Unit cell parameters determined from the different indexing methods and numbers of frames tested.

```
Refined crystal models:
model 1 (289 reflections):
Crystal:
    Unit cell: 6.20119(11), 11.8969(3), 15.5632(9), 90.007(3), 90.014(4), 89.9929(16)
    Space group: P 1
    U matrix:  {{ 0.7931,  0.6091,  0.0028},
                { 0.6090, -0.7927, -0.0262},
                {-0.0137,  0.0225, -0.9997}}
    B matrix:  {{ 0.1613,  0.0000,  0.0000},
                {-0.0000,  0.0841,  0.0000},
                { 0.0000,  0.0000,  0.0643}}
    A = UB:    {{ 0.1279,  0.0512,  0.0002},
                { 0.0982, -0.0666, -0.0017},
                {-0.0022,  0.0019, -0.0642}}
+------------+-------------+---------------+-------------+
|   Imageset |   # indexed |   # unindexed |   % indexed |
|------------+-------------+---------------+-------------|
|          0 |         289 |            18 |        94.1 |
+------------+-------------+---------------+-------------+
```

Figure S12. DIALS refined crystal model output from indexing via the automated pipeline using the fft3d method on 50 frames obtained for well 025.

```
Refined crystal models:
model 1 (306 reflections):
Crystal:
    Unit cell: 6.20113(10), 11.8969(3), 15.5629(9), 90.000(3), 89.986(3), 89.9930(15)
    Space group: P 1
    U matrix:  {{-0.7934, -0.6087,  0.0027},
                {-0.6086,  0.7931, -0.0260},
                { 0.0137, -0.0223, -0.9997}}
    B matrix:  {{ 0.1613,  0.0000,  0.0000},
                {-0.0000,  0.0841,  0.0000},
                {-0.0000, -0.0000,  0.0643}}
    A = UB:    {{-0.1279, -0.0512,  0.0002},
                {-0.0982,  0.0667, -0.0017},
                { 0.0022, -0.0019, -0.0642}}
+------------+-------------+---------------+-------------+
|   Imageset |   # indexed |   # unindexed |   % indexed |
|------------+-------------+---------------+-------------|
|          0 |         306 |            19 |        94.2 |
+------------+-------------+---------------+-------------+
```

Figure S13. DIALS refined crystal model output from indexing via manual processing using the fft3d method on 50 frames obtained for well 025.

```
Refined crystal models:
model 1 (65 reflections):
Crystal:
    Unit cell: 6.2008(2), 11.8977(7), 15.562(2), 90.017(9), 89.986(7), 89.988(4)
    Space group: P 1
    U matrix:  {{ 0.7681,  0.6404, -0.0042},
                { 0.6402, -0.7680, -0.0153},
                {-0.0131,  0.0090, -0.9999}}
    B matrix:  {{ 0.1613,  0.0000,  0.0000},
                {-0.0000,  0.0841,  0.0000},
                {-0.0000,  0.0000,  0.0643}}
    A = UB:    {{ 0.1238,  0.0538, -0.0003},
                { 0.1033, -0.0646, -0.0010},
                {-0.0021,  0.0007, -0.0643}}
+------------+-------------+---------------+-------------+
|   Imageset |   # indexed |   # unindexed |   % indexed |
|------------+-------------+---------------+-------------|
|          0 |          65 |             2 |          97 |
+------------+-------------+---------------+-------------+
```

Figure S14. DIALS refined crystal model output from indexing via manual processing using the fft3d method on 10 frames obtained for well 025.

```
Refined crystal models:
model 1 (41 reflections):
Crystal:
    Unit cell: 6.2021(5), 11.8989(13), 15.559(4), 89.965(18), 89.990(15), 90.005(6)
    Space group: P 1
    U matrix:  {{-0.7665,  0.6422,  0.0086},
                {-0.6422, -0.7665,  0.0089},
                { 0.0123,  0.0013,  0.9999}}
    B matrix:  {{ 0.1612,  0.0000,  0.0000},
                { 0.0000,  0.0840,  0.0000},
                {-0.0000, -0.0001,  0.0643}}
    A = UB:    {{-0.1236,  0.0540,  0.0006},
                {-0.1036, -0.0644,  0.0006},
                { 0.0020,  0.0001,  0.0643}}
+------------+-------------+---------------+-------------+
|   Imageset |   # indexed |   # unindexed |   % indexed |
|------------+-------------+---------------+-------------|
|          0 |          41 |             0 |         100 |
+------------+-------------+---------------+-------------+
```

Figure S15. DIALS refined crystal model output from indexing via manual processing using the fft3d method on 5 frames obtained for well 025.

```
Refined crystal models:
model 1 (24 reflections):
Crystal:
    Unit cell: 6.2008(7), 11.891(2), 15.554(6), 90.05(3), 89.91(3), 90.004(10)
    Space group: P 1
    U matrix:  {{-0.8448,  0.5342, -0.0315},
                {-0.5350, -0.8418,  0.0719},
                { 0.0119,  0.0775,  0.9969}}
    B matrix:  {{ 0.1613,  0.0000,  0.0000},
                { 0.0000,  0.0841,  0.0000},
                {-0.0002,  0.0001,  0.0643}}
    A = UB:    {{-0.1362,  0.0449, -0.0020},
                {-0.0863, -0.0708,  0.0046},
                { 0.0017,  0.0066,  0.0641}}
+------------+------------+--------------+------------+
|   Imageset |   # indexed |   # unindexed |   % indexed |
|------------+------------+--------------+------------|
|          0 |          24 |             2 |        92.3 |
+------------+------------+--------------+------------+
```

Figure S16. DIALS refined crystal model output from indexing via manual processing using the fft3d method on 3 frames obtained for well 025.

```
Refined crystal models:
model 1 (289 reflections):
Crystal:
    Unit cell: 6.20151(11), 11.8976(3), 15.5647(9), 90.0, 90.0, 90.0
    Space group: P 2 2 2
    U matrix:  {{-0.7934,  0.6087, -0.0024},
                {-0.6086, -0.7931,  0.0259},
                { 0.0139,  0.0220,  0.9997}}
    B matrix:  {{ 0.1613,  0.0000,  0.0000},
                { 0.0000,  0.0841,  0.0000},
                { 0.0000,  0.0000,  0.0642}}
    A = UB:    {{-0.1279,  0.0512, -0.0002},
                {-0.0981, -0.0667,  0.0017},
                { 0.0022,  0.0018,  0.0642}}
+------------+------------+--------------+------------+
|   Imageset |   # indexed |   # unindexed | % indexed   |
|------------+------------+--------------+------------|
|          0 |         289 |            18 | 94.1%       |
+------------+------------+--------------+------------+
```

Figure S17. DIALS refined crystal model output from indexing via the automated pipeline using the real_space_grid_search method on 50 frames obtained for well 025.

```
Refined crystal models:
model 1 (306 reflections):
Crystal:
    Unit cell: 6.20144(10), 11.8976(3), 15.5647(9), 90.0, 90.0, 90.0
    Space group: P n n m
    U matrix:  {{-0.7934,  0.6087, -0.0025},
                {-0.6086, -0.7931,  0.0260},
                { 0.0139,  0.0221,  0.9997}}
    B matrix:  {{ 0.1613,  0.0000,  0.0000},
                { 0.0000,  0.0841,  0.0000},
                { 0.0000,  0.0000,  0.0642}}
    A = UB:    {{-0.1279,  0.0512, -0.0002},
                {-0.0981, -0.0667,  0.0017},
                { 0.0022,  0.0019,  0.0642}}
+------------+------------+--------------+------------+
|   Imageset |   # indexed |   # unindexed |   % indexed |
|------------+------------+--------------+------------|
|          0 |         306 |            19 |        94.2 |
+------------+------------+--------------+------------+
Saving refined experiments to indexed.expt
Saving refined reflections to indexed.refl
```

Figure S18. DIALS refined crystal model output from indexing via manual processing using the real_space_grid_search method on 50 frames obtained for well 025.

```
Refined crystal models:
model 1 (24 reflections):
Crystal:
    Unit cell: 6.2067(7), 11.902(2), 15.593(6), 90.0, 90.0, 90.0
    Space group: P n n m
    U matrix:  {{-0.7668,  0.6418, -0.0154},
                {-0.6418, -0.7658,  0.0420},
                { 0.0152,  0.0421,  0.9990}}
    B matrix:  {{ 0.1611,  0.0000,  0.0000},
                {-0.0000,  0.0840,  0.0000},
                { 0.0000,  0.0000,  0.0641}}
    A = UB:    {{-0.1235,  0.0539, -0.0010},
                {-0.1034, -0.0643,  0.0027},
                { 0.0024,  0.0035,  0.0641}}
------------+------------+--------------+------------+
   Imageset |   # indexed |   # unindexed |   % indexed |
------------+------------+--------------+------------|
          0 |          24 |             2 |        92.3 |
------------+------------+--------------+------------+
```

Figure S19. DIALS refined crystal model output from indexing via manual processing using the real_space_grid_search method on 3 frames obtained for well 025.

Supplementary References

1 Winter, G. *et al.* DIALS: implementation and evaluation of a new integration package. *Acta Crystallographica Section D* **74**, 85-97, doi:10.1107/S2059798317017235 (2018).

2 Sheldrick, G. SHELXT - Integrated space-group and crystal-structure determination. *Acta Crystallographica Section A* **71**, 3-8, doi:10.1107/S2053273314026370 (2015).

3 Sheldrick, G. Crystal structure refinement with SHELXL. *Acta Crystallographica Section C* **71**, 3-8, doi:10.1107/S2053229614024218 (2015).

4 Dolomanov, O. V., Bourhis, L. J., Gildea, R. J., Howard, J. A. K. & Puschmann, H. OLEX2: a complete structure solution, refinement and analysis program. *Journal of Applied Crystallography* **42**, 339-341, doi:10.1107/S0021889808042726 (2009).

5 Diederichs, K. & Wang, M. in Protein Crystallography: Methods and Protocols (eds Wlodawer, A., Dauter, Z. & Jaskolski, M.) 239–272 (Humana Press, New York, 2017).

6 Weiss, M. S. & Hilgenfeld, R. On the use of the mergingRfactor as a quality indicator for X-ray data. *Journal of Applied Crystallography* **30**, 203-205, doi:10.1107/s0021889897003907 (1997).

7 Wang, J., Brudvig, G. W., Batista, V. S. & Moore, P. B. On the relationship between cumulative correlation coefficients and the quality of crystallographic data sets. *Protein Science* **26**, 2410-2416, doi:10.1002/pro.3314 (2017).

8 Karplus, P. A. & Diederichs, K. Assessing and maximizing data quality in macromolecular crystallography. *Current Opinion in Structural Biology* **34**, 60-68, doi:10.1016/j.sbi.2015.07.003 (2015).
