# Supplementary material for: Small-rotative fixed-target serial synchrotron crystallography (SR-FT-SSX) for molecular crystals
Source: Commun Chem. 2024 Nov 13;7:264. doi: 10.1038/s42004-024-01360-7 (PMC11561299; doi:10.1038/s42004-024-01360-7)
Supplement: Supplementary file 3 — Description of Additional Supplementary Files [file 42004_2024_1360_MOESM3_ESM.pdf]

Description of Additional Supplementary Files

File name: Supplementary Movie 1

Description: A video showing the small rotation of the fixed target serial grid, in-situ at the data collection positions on Beamline I19 at Diamond Light Source.
